# Optimization of the Extraction Protocol for Pacific Ciguatoxins from Marine Products Prior to Analysis Using the Neuroblastoma Cell-Based Assay

**DOI:** 10.3390/md23010042

**Published:** 2025-01-16

**Authors:** Thomas Yon, Philippe Cruchet, Jérôme Viallon, J. Sam Murray, Emillie Passfield, Mireille Chinain, Hélène Taiana Darius, Mélanie Roué

**Affiliations:** 1 Institut Louis Malardé (ILM), UMR 241-SECOPOL (IFREMER, ILM, IRD, UPF), Laboratory of Marine Biotoxins, P.O. Box 30, 98713 Papeete, Tahiti, French Polynesia; tyon@ilm.pf (T.Y.); pcruchet@ilm.pf (P.C.); jviallon@ilm.pf (J.V.); mchinain@ilm.pf (M.C.); 2 Cawthron Institute, 98 Halifax St East, PB 2, The Wood, Nelson 7010, New Zealand; sam.murray@cawthron.org.nz (J.S.M.); emillie.passfield@cawthron.org.nz (E.P.); 3 Institut de Recherche pour le Développement (IRD), UMR 241-SECOPOL (IFREMER, ILM, IRD, UPF), P.O. Box 6570, 98702 Faa’a, Tahiti, French Polynesia

**Keywords:** ciguatera poisoning, ciguatoxins, marine products, extraction protocol, CBA-N2a, LC-MS/MS, matrix effect, risk assessment, food safety

## Abstract

Ciguatera poisoning (CP) is caused by the consumption of marine products contaminated with ciguatoxins (CTXs) produced by dinoflagellates of the genus *Gambierdiscus*. Analytical methods for CTXs, involving the extraction/purification of trace quantities of CTXs from complex matrices, are numerous in the literature. However, little information on their effectiveness for nonpolar CTXs is available, yet these congeners, contributing to the risk of CP, are required for the establishment of effective food safety monitoring programs. An evaluation of six extraction/purification protocols, performed with CTX3C spiked on fish flesh and a neuroblastoma cell-based assay (CBA-N2a), revealed recoveries from 6 to 45%. This led to the development of an optimized 3-day protocol designed for a large number of samples, with CTX1B and CTX3C eluting in a single fraction and showing recoveries of 73% and 70%, respectively. In addition, a reduction in adverse matrix effects in the CBA-N2a analyses was demonstrated with naturally contaminated specimens, increasing the sensitivity of the method, which now meets the very low guidance level recommended by international agencies. However, efforts are still required to reduce the signal suppression observed in LC-MS/MS analysis. This optimized protocol contributes to the technological advancement of detection methods, promoting food safety and improving CP risk assessment in marine products.

## 1. Introduction

Ciguatera poisoning (CP) is a foodborne illness caused by the consumption of marine products (fish or invertebrates) contaminated with ciguatoxins (CTXs), potent neurotoxins produced by certain species of dinoflagellates of the genus *Gambierdiscus* [1,2]. CP is prevalent in all tropical and subtropical regions, including islands in the Caribbean, Pacific and Indian Oceans, as well as coastal areas of the Americas, Africa and Asia [3]. While the true incidence of CP worldwide is difficult to estimate due to underreporting and occasional misdiagnosis, this poisoning is considered the most common marine toxin-related illness [3].

The toxins responsible for CP, i.e., CTXs, are a suite of lipid-soluble polyethers that are bioaccumulated and metabolized through the food chain from herbivores to carnivores (shellfish or fish). This results in a large class of biotransformed analogues with varying degrees of oxidation, saturation and/or ring systems opening [4,5]. Depending on their structure and polarity, CTXs exert different effects on voltage-gated sodium channels (VGSCs) in excitable cells, leading to alterations in neuronal function and neurotransmitter release [6]. Hence, the symptoms and severity observed in CP cases largely depend on an individual’s medical history and sensitivity, as well as the section and amount of the marine specimen consumed [7,8]. A variety of symptoms can manifest, generally starting with gastrointestinal (e.g., nausea, vomiting, diarrhea), followed by cardiovascular (e.g., bradycardia, hypotension) and then neurological (e.g., paresthesia, dysesthesia, pruritus, cold allodynia, etc.) [9]. The onset of symptoms typically occurs within hours to days after the ingestion of the contaminated marine product and may persist for weeks to years in some cases [9]. Identifying CP cases can be challenging due to their large variety of symptoms and the lack of specific clinical diagnostic tests. Diagnosis relies on a thorough history of fish consumption by the patient presenting evocative symptoms following the ingestion of reef fish [10]. Confirmation of CP requires analytical methods able to detect the presence of CTXs in leftover meal remnants; however, these are seldomly available. The very low level of CTXs in marine products, which is nonetheless sufficient to trigger symptoms in humans due to their very high toxicity (i.e., 0.01 µg kg^−1^ body weight [11]), makes their detection in leftover meal remnants very difficult [12].

Only a few approaches are sufficiently sensitive to detect CTXs in trace amounts, such as the mouse neuroblastoma cell-based assay (CBA-N2a) [13,14,15], the receptor binding assay [16,17,18,19], sandwich ELISA detection using monoclonal antibodies [20,21,22] and liquid chromatography coupled to tandem mass spectrometry (LC-MS/MS) [23,24,25,26,27]. In addition, the lack of certified reference materials (both analytical standards and contaminated marine products) severely hampers the development and validation of detection methods [28]. In terms of food safety issues, experts recommend that ciguatera risk management programs be conducted using a two-tiered analysis approach [11]. This typically consists of using sensitive functional methods for the screening and quantification of biological samples, such as the CBA-N2a, followed by the confirmation of CTX analogues in toxic samples using specific analytical methods, such as the LC-MS/MS.

Although these detection methods are based on very different measurement mechanisms, i.e., mode of action or chemical structure, a key step in each of these approaches is sample preparation, which must ensure the most efficient extraction of all CTXs from marine product tissues and lead to a purified fraction that can be analyzed directly without interference [29]. This represents a major challenge because only trace quantities of CTXs are accumulated in marine products (typically in the µg kg^−1^ range), which are very complex matrices rich in lipids and proteins, compounds that often co-elute with CTXs and may be responsible for matrix effects inherent to the detection method used. Therefore, numerous sample preparation protocols have been described in the literature, comprising similar main stages, i.e., extraction, liquid–liquid partitioning and solid phase extraction (SPE) clean-up steps, but each with different conditions (see Harwood et al. [28] for a review; [23,24,30]). The majority of these protocols have been developed for the more polar CTXs found primarily in carnivorous fish—such as CTX1B, 52-epi-54-deoxyCTX1B and 54-deoxyCTX1B (also called CTX2 and CTX3, respectively) or 2,3-dihydroxyCTX3C—, for which recoveries ranging from 26 to 96% have been reported, depending on the sample preparation protocol and detection method used, but also on the marine product analyzed (Table 1). In contrast, their efficiency for less polar analogues found throughout the trophic chain—such as CTX3B, CTX3C, CTX4A or CTX4B—have rarely been evaluated, and when they are, very low recoveries have been reported, ranging from 13 to 35%, except for a recent study reporting a recovery of 107% (Table 1). However, these nonpolar CTXs contribute to the risk of CP in the Pacific as several poisonings have been reported following the consumption of herbivorous fish or invertebrates, which mostly contain these nonpolar congeners [31,32,33,34,35,36,37]. Thus, these often-reported low recoveries imply a risk of underestimating the potentially significant quantities of CTXs in marine products with potential deleterious consequences for food safety and thus the public health of consumers. In addition, most sample preparation protocols involve multiple steps (up to five) and are, therefore, relatively time-consuming, making them incompatible with the characterization of the ciguatera risk of an area by analyzing a large number of samples [38,39,40].

In this context, to obtain a more reliable assessment of the actual levels of CTXs accumulated in marine products, the present study aimed to work on an extraction/purification protocol that would i) optimize the recovery of the nonpolar CTXs, ii) produce an extract with reduced adverse matrix effects observed in CBA-N2a experiments and iii) have a simple and short execution time to make it applicable for the screening of a large number of samples. First, the efficiency of six sample preparation protocols, inspired by some available in the literature, was evaluated on fish flesh spiked with a CTX3C standard and analyzed by the CBA-N2a. Second, based on the results obtained, an improved extraction/purification protocol was then developed for the subsequent detection and quantification of CTXs by the CBA-N2a, regardless of their polarity. Third, the performance of this new protocol was evaluated for the subsequent detection of CTXs by LC-MS/MS.

## 2. Results

### 2.1. Evaluation of Efficiencies of Six Different Protocols for the Extraction/Purification of CTX3C

The importance of the parameters used in protocols for CTX extraction from marine products was studied using six different protocols inspired by those available in the literature (protocols #1 to #6, see Section 4.5 and Supplementary Materials). In an initial step, the six protocols were applied to a non-toxic fish sample (10 g), the Tuamotu emperor *Lethrinus atkinsoni* (Lethrinidae), to determine the matrix effects observed on N2a cell viability. Fractions of interest (i.e., the fraction expected to contain the majority of the CTXs and thus used for subsequent analysis) but also washing fractions having an extract dry mass less than 30 mg were analyzed to determine the maximum concentration of dry extract (MCE) and its equivalent in wet fish flesh (Table 2) that can be tested without causing adverse matrix effects (see Section 4.8.2).

A significant difference in MCE values was observed across the protocols: in the cases of protocols #1 and #2, for the fraction of interest, a concentration of 34 and 84 mg eq. wet weight of fish flesh, respectively, resulted in the non-specific mortality of N2a cells, whereas the maximal concentration tested for protocols #3, #4 and #5 did not induce any mortality (Table 2).

Next, three CTX3C-spiked flesh samples of the same Tuamotu emperor (5 ng CTX3C in 10 g wet weight of fish flesh) were extracted and purified according to each protocol. The fractions obtained were first screened by the CBA-N2a in a single dose at the MCE to identify and eliminate negative fractions (no mortality in OV- and OV+ conditions) from the analyses. Then, CTX quantification was performed only in fractions showing CTX-like activity (mortality in OV^+^ condition only) using an appropriate range of concentrations to obtain the EC_50_ value and calculate the quantity of CTX3C actually recovered, thus allowing the evaluation of the nonpolar CTX extraction efficiency from 5 ng spiked samples.

The CBA-N2a was able to quantify very low quantities of CTX3C from 0.13 ± 0.01 up to 2.11 ± 0.18 ng CTX3C eq. with good reproducibility. The coefficients of variation (CVs) ranged from 7.5 to 23.3%, except for the fraction F3.1 from protocol #4 tested at 0.15 ± 0.09 ng CTX3C eq., which showed a CV of 55.9% (Table 2). This result can be attributed to the difficulty of extracting/analyzing trace amounts of CTXs, which usually results in higher variability. In terms of percentage of CTX3C recovery, this corresponded to a minimum of 6.4 ± 0.9 and a maximum of 45.3 ± 1.0%. The conditions resulting in the highest recoveries of spiked CTX3C were from protocols #1, #4 and #6 (Table 2), in which the extraction solvent was either acetone, methanol (MeOH)/Hexane 73:27 or MeOH/H_2_O 90:10, whereas extractions with MeOH/H_2_O 60:40 or a two-step extraction with first MeOH 100% and then MeOH/H_2_O 60:40 resulted in less than 25% recoveries of CTX3C (protocols #2 and #5).

In addition, using protocols #1, #3, #4 and #5, CTX3C was only partially recovered or not recovered at all in the fraction of interest (Table 2). Indeed, for protocols #1 and #3, a distribution of CTX3C in two different fractions obtained after the final SPE (C18) was observed, i.e., with 7.6 ± 1.2 and 21.5 ± 3.1% of CTX3C spike recovery in the fraction of interest, and 10.7 ± 1.9 and 15.4 ± 3.2% in the washing fraction, respectively. Furthermore, for protocols #4 and #5, the extracted CTX3C was only eluted in the additional washing phase of the C18 SPE step, resulting in the complete absence of CTX3C in the fraction of interest.

Finally, from a purely technical point of view, protocol #2 proved to be the fastest and simplest, while protocol #6 proved to be the most complicated one, with five purification steps, and protocol #1 was the most time-consuming, requiring eight days to complete.

### 2.2. Design of an Improved Protocol for CTX Extraction and Purification

**Figure 1:**
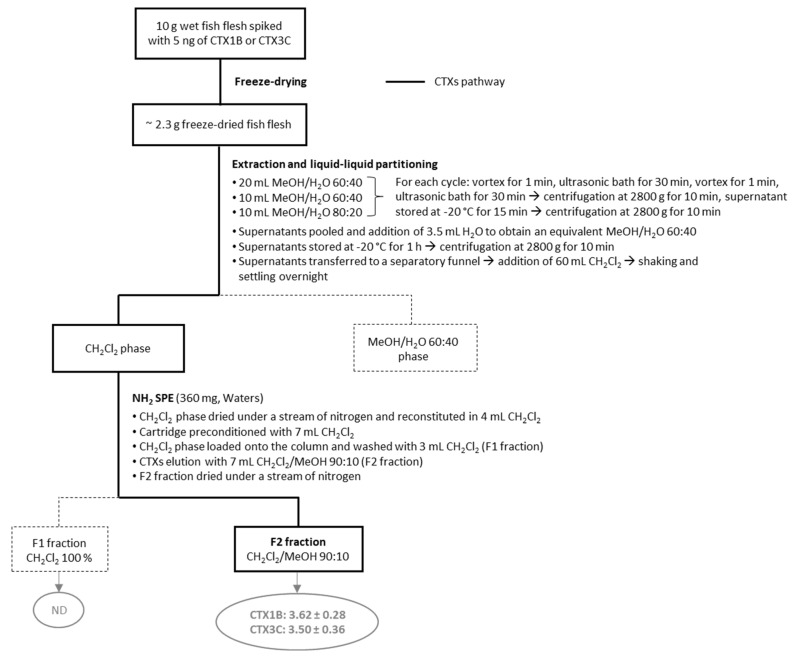
Schematic representation of the OP protocol with CTX1B and CTX3C amounts (ng) estimated by the CBA-N2a in testable fractions. Data represent the mean ± standard deviation (SD) (each concentration run in triplicate wells) of three independent experiments run on different days. In bold is the CTX pathway through the different steps.

Building on the positive attributes of each of the six protocols previously evaluated, preliminary assays on fish flesh samples spiked with CTX1B or CTX3C standards led to the development of an optimized protocol (named OP), shown in Figure 1.

Firstly, the wet fish flesh was freeze-dried to overcome differences in water content between the different samples and to facilitate the shipment and exchange of the samples between laboratories. The percentage of aqueous MeOH used in the extraction step was optimized to improve the recovery of the nonpolar CTXs. Briefly, four conditions (i.e., MeOH/H_2_O 60:40, MeOH/H_2_O 80:20, MeOH 100% and dichloromethane (CH_2_Cl_2_) 100%) were tested on spiked fish flesh and the best compromise between CTX3C recovery and a low amount of matrix co-extractives was obtained using MeOH/H_2_O 80:20.

Furthermore, to reduce the number of evaporation/redissolving steps on the extracts, and as proposed in protocol #2, a liquid/liquid partitioning step (MeOH/H_2_O 60:40 vs. CH_2_Cl_2_ 100%) was performed twice directly using the supernatant obtained from the extraction step.

Finally, based on the results previously obtained with protocol #2 (see Section 2.1), an aminopropyl (NH_2_) SPE column was used to recover both polar and nonpolar CTXs in a single fraction.

This new improved protocol thus allowed CTX1B and CTX3C standards to be eluted in a single fraction of interest (F2 fraction), with recoveries of 72.5 ± 5.7 and 70.0 ± 7.2% (n = 9) for CTX1B and CTX3C, respectively, as estimated by the CBA-N2a, and with a completion time of three days.

### 2.3. Application of the OP Protocol to Naturally Contaminated Marine Product Samples

To confirm the efficiency of the improved OP protocol, additional tests were performed on naturally contaminated herbivorous and carnivorous marine products samples collected from French Polynesian ciguateric areas. For comparison purposes, the samples were extracted in parallel using both protocol #1 (already used in previous studies to analyze French Polynesian marine products samples) and the OP protocol. Then, for each marine product, the fraction of interest obtained from the two protocols (i.e., the F2 fraction eluted from a C18 SPE cartridge with MeOH/H_2_O 90:10 for protocol #1, and the F2 fraction eluted from an NH2 SPE cartridge with CH_2_Cl_2_/MeOH 90:10 for the OP protocol) were tested in parallel using the CBA-N2a to determine their respective CTX contents.

**Figure 2:**
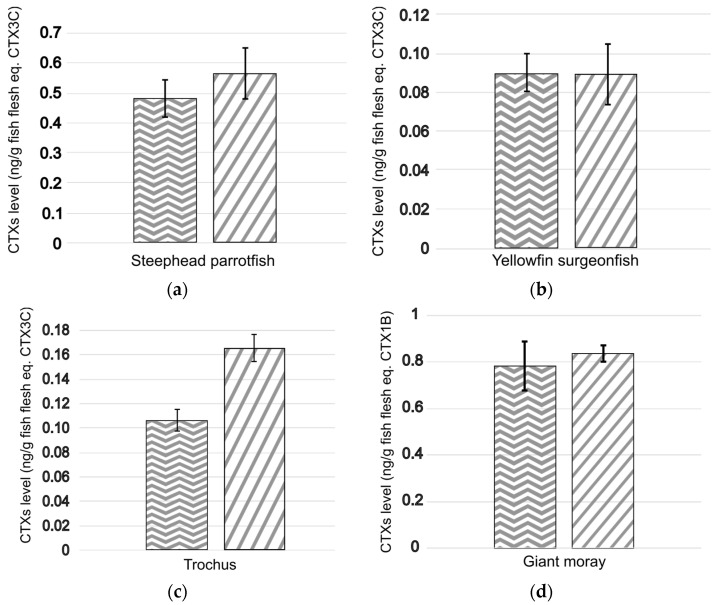

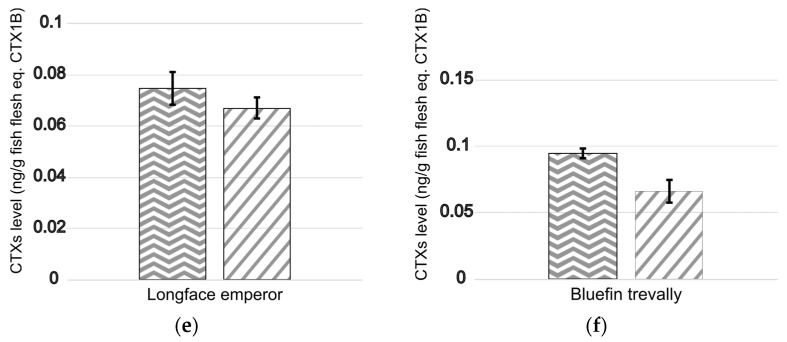
CTX levels estimated with the CBA-N2a in fractions of interest purified from naturally contaminated fish and shellfish using protocol #1 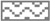 and the OP protocol 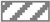. Data represent the mean ± SD (each concentration run in triplicate wells) of three independent experiments run on different days on at least two extraction replicates. The marine products analyzed were for the herbivores (**a**) steephead parrotfish, *Chlorurus microrhinos*, (**b**) yellowfin surgeonfish, *Acanthurus xanthopterus*, and (**c**) trochus, *Tectus niloticus*, and the carnivores (**d**) giant moray, *Gymnothorax javanicus*, (**e**) longface emperor, *Lethrinus olivaceus*, and (**f**) bluefin trevally, *Caranx melampygus*.

The results showed that the estimated CTX levels for carnivorous fish (likely to contain mostly polar CTXs) were broadly similar for both protocols, ranging from 0.075 ± 0.006 to 0.714 ± 0.103 and from 0.067 ± 0.004 to 0.836 ± 0.036 ng eq. CTX1B g^−1^ wet fish flesh for protocol #1 (n = 9) and the OP protocol (n = 6), respectively (Figure 2). Equivalent toxicities were also calculated for the two herbivorous fish samples (likely to contain mostly nonpolar CTXs), with CTX levels quantified at 0.48 ± 0.06 and 0.09 ± 0.01 ng CTX3C eq. g^−1^ wet fish flesh for protocol #1 (n = 9) and 0.57 ± 0.09 and 0.09 ± 0.02 ng CTX3C eq. g^−1^ wet fish flesh for the OP protocol (n = 6) in the steephead parrotfish *Chlorurus microrhinos* and yellowfin surgeonfish (*Acanthurus xanthopterus*) samples, respectively (Figure 2). However, regarding the trochus *Tectus niloticus*, a 1.6-fold increase in the CTX level measured was observed using the OP protocol (16.6 ± 1.1 ng CTX3C eq. g^−1^ wet flesh) compared to protocol #1 (10.6 ± 0.9 ng CTX3C eq. g^−1^ wet flesh) (Figure 2).

**Figure 3:**
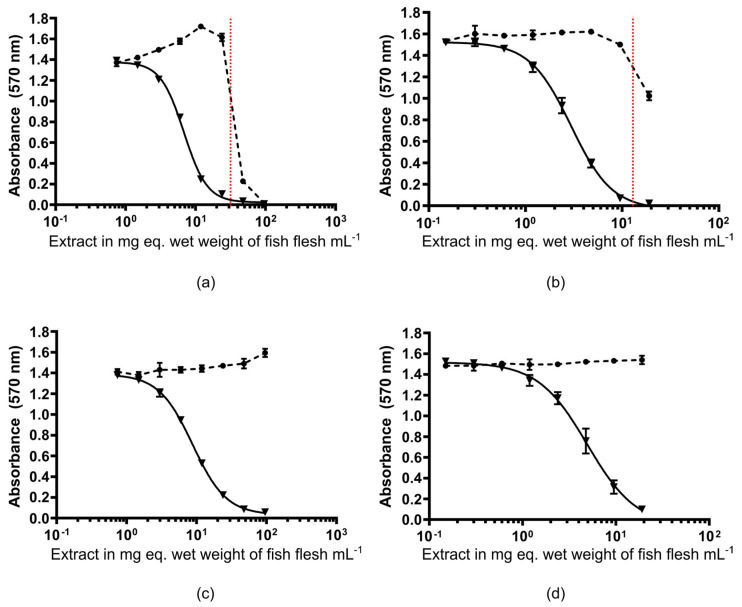
Cytotoxic dose–response curves under OV^-^ (black circle) and OV^+^ (black triangle) conditions obtained using the CBA-N2a on fractions of interest purified from naturally contaminated yellowfin surgeonfish and bluefin trevally samples using protocol #1 (**a**,**b**) and the OP protocol (**c**,**d**). Data represent the mean ± SD of each concentration run in triplicate wells. Non-specific mortality due to matrix effects is observed above the dotted red line.

The OP protocol also removed the adverse matrix effects previously observed, as demonstrated by the absence of non-specific mortality in the CBA-N2a at the maximum concentration tested (100 mg equivalent wet weight per mL) for the yellowfin surgeonfish and bluefin trevally samples (Figure 3). This reduction in matrix effects enabled the analysis of more than 100 mg eq. wet weight mL^−1^ with the new OP protocol versus. 34 mg eq. wet weight mL^−1^ with protocol #1 (Table 2). This provided improved limit of detection (LOD) and limit of quantification (LOQ) values for the CBA-N2a by 3-fold compared to the values published in Viallon et al. [13], now estimated at 0.01 and 0.02 ng CTX3C eq. g^−1^, respectively. Of note, the improved LOD and LOQ meet the guidance safety limits recommended by the US-FDA and EFSA [50,51].

### 2.4. Evaluation of the Suitability of the OP Protocol for LC-MS/MS Sample Analyses

In view of the promising results obtained with the CBA-N2a, the suitability of the OP protocol for LC-MS/MS sample analyses was subsequently evaluated.

Since the recoveries calculated from the LC-MS/MS analyses were consistently much lower than those obtained with the CBA-N2a, fortification experiments were performed to evaluate the matrix effects using two carnivorous fish and one herbivorous fish sample, i.e., a spiking of the final fraction of interest with two mixes of CTX1B, CTX3B, CTX3C and CTX4A, with each standard at a final concentration of 5 or 10 ng mL^−1^.

**Figure 4:**
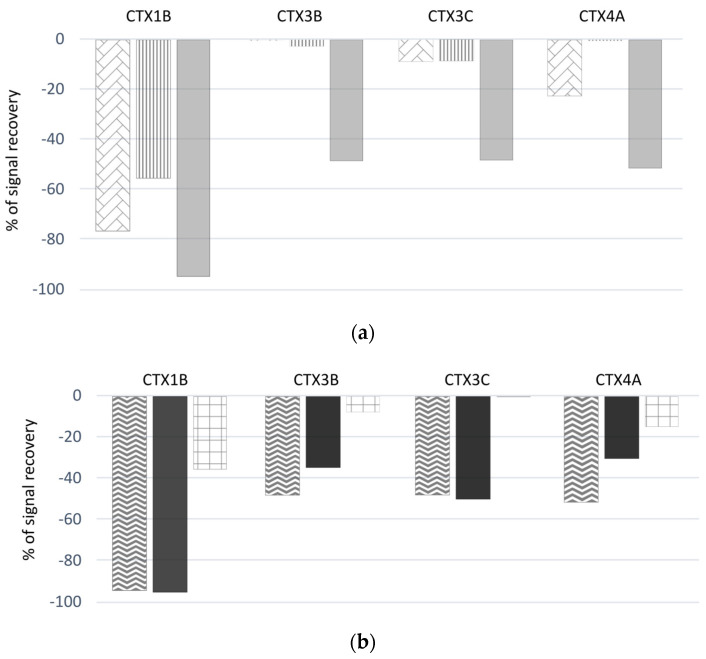
Percentages of signal suppression observed in LC-MS/MS analysis after fortification experiments (spiking extracts with a mix of CTX1B, CTX3B, CTX3C and CTX4A standard solutions) of the (**a**) steephead parrotfish 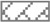, marbled grouper 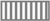 and bluefin trevally 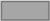, obtained with the OP protocol, as well as of the (**b**) bluefin trevally, obtained with the OP protocol 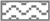, protocol #1 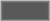 and the protocol published in Murray et al. [27] 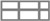.

Significant matrix effects were observed when analyzing the extracts of the three fishes generated using the OP protocol (Figure 4a). First, varying LC-MS/MS signal suppression was observed, which was species-specific and showed that the bluefin trevally sample induced the highest suppression. Practically, up to 95% signal suppression was evident for CTX1B for this sample, while ranging from 52 to 59% for CTX3B, CTX3C and CTX4A. Overall, CTX1B was the analogue most affected by the matrix signal suppression, with a suppression effect of 58–95% depending on the fish species. For nonpolar ciguatoxins, signal suppression was low (<25%) in the parrotfish and grouper samples.

In addition, the same bluefin trevally sample was extracted using the OP protocol, protocol #1 and the protocol described in Murray et al. [27] (from which protocol #2 was inspired) (Figure 4b). Using protocol #1, equivalent signal suppressions were observed for the fractions of interest as compared to the OP protocol. In contrast, the fractions of interest purified using the Murray et al. [27] protocol, which was developed for LC-MS/MS analysis, showed much lower signal suppressions (i.e., 36% for CTX1B; 8% for CTX3B; 1% for CTX3C; and 15% for CTX4A) compared to the OP protocol.

## 3. Discussion

The globalization of CP has led to renewed interest from the scientific community and regulatory agencies with regard to the management of this prominent public health issue. As a result, experts have identified several priority areas for improvement, one of which is the optimization of toxin detection in marine products [11,52]. Indeed, the implementation of efficient CP risk surveillance and food safety monitoring programs is currently hampered by the critical need to improve the analytical methods used for CTXs, including sample preparation protocols [28]. When it comes to the detection and quantification of CTXs in marine products, the challenge is considerable due to (i) the trace amounts of CTXs usually present in marine product samples, though sufficient to trigger ciguatera food poisoning [12], and (ii) the complexity of these matrices, whose compositions also vary according to the part of the organism analyzed, species, size, time of year, fishing site and geographic origin [53].

Hence, achieving the development of rapid and effective protocols for extracting and purifying CTXs from marine products is critical for the development of effective monitoring programs, which will ultimately reduce the health risk to communities. Several approaches have been reported in the literature, involving sample sizes ranging from 1 to 10 g of wet flesh or a freeze-dried equivalent, as well as an extraction step (preceded or not by a cooking step or enzymatic digestion) with organic solvents or a mixture of organic and aqueous solvents, followed by one or more purification steps, either by liquid–liquid partitioning or by SPE, to reduce the matrix co-extractives among the CTXs [23,24,37,54]. However, comparing the performance of these protocols remains difficult, if not impossible, due to the lack of standardized protocols across laboratories/studies [11] and the lack of a common source of reference standards.

The aim of the present study was therefore to design a simple and rapid extraction/purification protocol applicable to the high-throughput screening of a large number of samples, while ensuring the recovery of at least 70% of all CTXs present (regardless of their polarity) and a reduction in matrix effects to improve CBA-N2a sensitivity.

### 3.1. CTX3C Extraction Efficiency

In this study, CTX3C recovery yields were compared between the various protocols available from the literature using triplicates of spiked fish with a single batch of standards also used for CBA-N2a analyses, enabling a direct and reliable comparison of the parameters used at each stage of these protocols. Studies using a similar approach are scarce in the literature [23,24,27] due to the limited availability and high cost of reference standards but was made possible in the present study using the standards available from the bank of CTX standards of the Institut Louis Malardé (ILM).

The results showed that the six protocols evaluated were poorly suited to the extraction and purification of nonpolar CTXs. In fact, CTX3C recoveries ranging from 0 to 41% were found in the final fractions of interest, with the remainder of the CTX3C either not extracted from the fish flesh or found in the other fractions. In protocol #2 (inspired by Murray et al. [27]), a low CTX3C recovery of 6% was observed, consistent with their study, evaluating a CTX3C recovery of 13%. In contrast, with protocol #6 (inspired by Nagae et al. [23]), a 41% CTX recovery was found in the present study while a recovery above 100% was reported by the authors. Nevertheless, this represents the best CTX3C recovery of the six protocols evaluated. The lower nonpolar CTX recovery rate observed in the present study could be due to the difficulty of reproducing the complex six-step sample preparation protocol proposed by Nagae et al. [23]. The four other protocols, from which protocols #1, #3, #4 and #5 were inspired, did not previously evaluate the recovery of nonpolar CTXs [25,45,55,56], but reported satisfactory recoveries for polar CTXs, confirming their suitability for the extraction/purification of this group of CTX analogues. For example, Mak et al. [45], from which protocol #5 was inspired, reported recoveries ranging from 61 to 87% for CTX1B, CTX2 and CTX3. Meyer et al. [56], from which protocol #3 was inspired, demonstrated a 46% improvement in CTX1B recovery compared to the protocol of Lewis et al. [42], which previously reported a CTX1B recovery of 85%. Finally, Nagae et al. [23] reported CTX1B recoveries of around 90%. However, it is important to note that some of these studies used matrix-corrected standards (i.e., considering the potential matrix effect of the extract analyzed) for their quantification compared with the solvent standards, resulting in a significant impact on their reported recovery percentages.

In addition, although most of the methods reported sensitivities meeting CTX sanitary thresholds [50,51] and could therefore detect the presence of CTXs in low-level contaminated marine products, it is likely the actual quantity of CTXs per g of wet flesh reported was greatly underestimated, which could lead to the misclassification of fish as non-toxic or safe for consumption.

All of these observations suggest that there is potential for a significant improvement to most of the currently published protocols, especially when it comes to the increased recovery of all the CTXs present in a single fraction of interest, regardless of their polarity.

### 3.2. Finding the Best Compromise Between Extraction Efficiencies of Ciguatoxins and Matrix

The work carried out in the present study helped provide useful insights into some of the protocol parameters likely to influence the recovery efficiency of nonpolar CTXs such as CTX3C.

One of these parameters relates to the choice of extraction solvent. The results obtained for protocols #3 and #4 highlighted that the use of a pure organic solvent such as methanol or acetone without the addition of water was not efficient for the extraction of nonpolar CTXs from freeze-dried flesh samples. Indeed, although protocols #1 and #3 shared the same extraction method (i.e., acetone, ultrasonic bath), the percentage of recovery of CTX3C with protocol #3 (23%) was lower than with protocol #1 (32.2%) because protocol #3 was carried out on freeze-dried fish flesh as opposed to the wet fish flesh (containing approximately 80% of water) in protocol #1, as previously suggested by Lewis et al. [42]. From a technical perspective, it is likely that the presence of water in the organic solvent/water mixture causes the freeze-dried flesh fibers to swell, thus increasing their contact surface with the solvent. Nevertheless, if the proportion of water in the organic solvent/water mixture selected for extraction is too high (i.e., >30%), there is a risk of reducing the extraction efficiency of nonpolar CTXs, as demonstrated for protocol #2.

The second parameter concerns the liquid–liquid partition step. Despite the different biphasic mixtures chosen (aqueous MeOH/CH_2_Cl_2_ for protocols #1 and #2; aqueous MeOH/Hexane for protocols #3, #4 and #6; and ACN/CH_2_Cl_2_ for protocol #5), low losses of CTX3C were observed at this step across all protocols, with only traces of CTXs detected in the wash phase.

The third parameter regards the SPE clean-up step to limit the amount of matrix co-extractives extracted with the CTXs [14]. The results obtained in this study highlighted the importance of the choice of the mobile phase used for CTX elution with C18 SPE. Indeed, when the concentration of H_2_O in the mobile phase (i.e., a MeOH/H_2_O mixture) exceeds 20%, a large amount of CTX3C remains on the solid phase, as demonstrated in protocol #4, consistent with previous findings by Spielmeyer et al. [24], whereas less CTX3C remains on the solid phase when 10% of H_2_O is used, as in protocols #1 and #3. This result suggests a very narrow range of H_2_O percentages should be used in an attempt to elute all CTX3C but without recovering too much matrix. Actually, when no H_2_O is added to the mobile phase, such as in protocol #6, the totality of the CTX3C is eluted; however, this may considerably limit the interest of this SPE step as numerous matrix compounds are likely eluted at the same time. Alternative SPEs can also be used, as in protocols #4 and #5, in which silica was used as the stationary phase. However, for these two protocols, since the nonpolar CTXs remained on the solid phase during the previous step, it was not possible to evaluate the true benefits of this silica stationary phase. Al-though the six protocols tested were primarily developed to ensure the elution of polar CTXs in the fraction of interest, the present study was useful in characterizing the elution conditions enabling polar and nonpolar CTX recovery in a single fraction: the only stationary phase allowing the recovery of both polar (as described in Murray et al. [27]) and nonpolar CTXs in the same fraction was aminopropyl-modified silica with MeOH/CH_2_Cl_2_ 90:10 for the elution.

Finally, another key factor to consider is the dry weight of the fraction of interest and related matrix effects. According to the CBA-N2a results, the purification steps of protocols #3, #4, #5 and #6 significantly reduced the matrix effects to a point where no matrix effects were observed. In contrast, non-specific mortality was observed in N2a cells when exposed to concentrations of 34 and 84 mg eq. wet weight of fish flesh, respectively, obtained with protocols #1 and #2, respectively. The use of several SPEs is therefore recommended to reduce these matrix effects; however, the results obtained in this study highlight the fact that these steps are also a potential source of a significant loss of nonpolar CTXs. The careful set-up of the mobile and stationary phase conditions is therefore of utmost importance to ensure the best compromise possible.

Overall, these findings indicated that there was potential for the design of an improved extraction/purification protocol for all polar and nonpolar CTXs simultaneously.

### 3.3. Improved Extraction/Purification Protocol for CTXs

The second objective of this study was to develop a high-throughput protocol that would combine the pros of the previously evaluated protocols, while ensuring the recovery of at least 70% of the polar and nonpolar CTXs in a single fraction.

To this end, two solvent phases were successively used for the extraction, first 60:40 MeOH/H_2_O, well suited for swelling the fish flesh fibers and extracting polar CTXs, followed by MeOH/H_2_O 80:20, more suited for the extraction of nonpolar CTXs while limiting the amount of lipid co-extractives in the resulting extract. This latter supernatant was then directly subjected to 60% aqueous MeOH/CH_2_Cl_2_ liquid–liquid partitioning, reducing the risk of losses during evaporation cycles. This liquid–liquid partitioning is a cost-effective step commonly used for CTX purification from *Gambierdiscus* extracts [57], and it allowed a reduction in the dry mas extract by 16-fold. In addition, the SPE aminopropyl-modified silica phase also helped reduce extract complexity, as illustrated by the reduction in the final extract dry mass by 3-fold. As a result, the newly designed protocol, which required only three days to complete, allowed the recovery of > 72% of the CTX1B and 70% of the CTX3C standards spiked on fish flesh in a single fraction of interest following two purification steps.

This new protocol shares many similarities with the protocol published in Murray et al. [27], most notably the liquid–liquid partitioning and aminopropyl SPE steps. However, the OP protocol suggests an additional extraction step to improve the yield of nonpolar CTXs. Furthermore, in contrast to Murray et al. [27], the whole extract is kept throughout the protocol, which improves sensitivity and reduces the risk of false negatives when analyzing marine products with low CTX contents. In the meantime, other methods have been published, such as the study by Spielmeyer et al. [24], presenting a rapid and innovative protocol, but with a distribution of CTX3C in two different fractions and less than 35% recovery of spiked CTX3C in the fraction of interest (named CTX3C–eluate). More recently, a shortened version of this protocol was proposed by Loeffler et al. [58], but as no validation by spiking experiments was performed, the actual recovery rates remain unknown, and thus the efficiency of this protocol could not be evaluated at this time. Another protocol was also proposed by Nagae et al. [23], which provides satisfactory extraction yields for polar and nonpolar CTXs, but whose complexity and cost (i.e., it involves two liquid–liquid partitionings and four SPE steps) make it unsuitable for the screening of a large number of samples.

### 3.4. Applicability of the OP Protocol to Naturally Contaminated Fish Samples

The applicability of the OP protocol was further evaluated on a selection of six naturally contaminated samples—three carnivorous fish, two herbivorous fish and one gastropod—collected in French Polynesia during field trips in known ciguateric areas [35].

Unlike the previous spiking experiments in which a single CTX standard was spiked onto fresh flesh prior to freeze-drying, evaluating the OP protocol directly on naturally contaminated fish provides an opportunity to study the efficiency of the protocol on more complex samples. Indeed, naturally contaminated samples contain CTXs that are actually embedded in their flesh, and whose toxin profiles are potentially more complex due to a possible metabolization of the CTXs bioaccumulated in organisms from lower trophic levels [5,37,59,60]. Due to the limited amount of naturally contaminated material, the OP protocol was only compared to protocol #1, which was used beforehand for the selection of naturally contaminated samples.

When applied to several marine products naturally contaminated with different concentrations of CTXs (between 0.07 and 0.8 ng CTX1B eq. g^−1^ for carnivorous fish, and between 0.09 and 0.5 ng CTX3C eq. g^−1^ for herbivorous fish and shellfish), the OP protocol confirmed its good performance. This was demonstrated by the higher CTX levels measured in the steephead parrotfish and trochus samples versus similar toxicities in the three carnivorous fish and the yellowfin surgeonfish when compared to the protocol #1 toxicity data. These results align with those obtained from our previous spiking experiments. Indeed, since the OP protocol had a better recovery of nonpolar CTXs compared to protocol #1, the quantity of CTXs in CTX3C equivalence increased due to the high proportion of nonpolar CTXs contained in the steephead parrotfish and trochus shell samples [25,33]. Similarly, since the OP protocol did not significantly improve the recovery of CTX1B, the CTXs’ quantity in CTX1B equivalence measured in the three carnivorous fish was similar between the two protocols.

For the herbivorous yellowfin surgeonfish sample, a similar toxicity was observed using both protocols. However, the OV- curve (i.e., without treatment sensitizing the cells to sodium channel activators such as CTXs) observed during the CBA-N2a analysis of the sample extracted with protocol #1 demonstrated an important matrix effect. Indeed, for sensitivity purposes, since the CTX amount in this sample was very low, a dry extract mass higher than the apparent MCE was analyzed for the fraction obtained with protocol #1, leading to matrix effects and thus to an overestimation of the CTX amount.

### 3.5. Matrix Effect Evaluation of the OP Protocol for Subsequent CBA-N2a and LC-MS/MS Analyses

One of the key considerations for a sensitive and robust analysis is to ensure that no potential matrix effect could interfere with the analysis, e.g., the non-specific mortality of N2a cells in the CBA-N2a. Indeed, as demonstrated for the yellowfin surgeonfish sample in the present study, this non-specific mortality can be observed above a certain concentration of fish dry extract, consistent with observations from previous studies [13,14,61,62]. Therefore, it is of utmost importance to i) ensure the sample preparation is well suited to limit such matrix effects and increase the test sensitivity and ii) determine the MCE prior to N2a cell exposure to the fish extracts (e.g., by performing a preliminary analysis on non-toxic fish or under OV- condition). For LC-MS/MS analysis, the matrix effects can be observed by an increase in background noise [23,56], but also by signal suppression phenomena [27,63], which can differ depending on the characteristics of the sample and the CTX analogues considered. This is why, depending on the study, the detection limits using LC-MS/MS may vary for each analogue (depending on the specific CTX relative response factor and signal suppression or enhancement effects from matrix co-extractives) [25]. The OP protocol demonstrated a low matrix effect in the CBA-N2a analysis with an MCE > 100 mg equivalent wet weight mL^−1^, which represents a true advancement for detecting low levels of CTXs. Unfortunately, it was not suitable for a subsequent detection of CTXs by LC-MS/MS, which was likely due to the residual presence of compounds that strongly interfered with CTX signals, particularly with polar CTX analogues (from 55 to 95% of signal suppression). In comparison, the protocol of Murray et al. [27] seemed to be more suitable for the detection of CTXs by LC-MS/MS, but on the other hand were less effective for their detection by the CBA-N2a. This suggests that the compounds interfering with LC-MS/MS analyses differ from those interfering with the CBA-N2a. The present results also highlight the impact of the matrix on the results of the final toxin profile obtained, and it seems very important when LC-MS/MS analyses are carried out to take into account this matrix effect, either using matrix-calibrated standards [64] or fortification experiments [27].

Risk surveillance and food safety monitoring programs of CP require the analysis of large numbers of samples in order to assess the levels of CTXs accumulated in marine products commonly consumed by local populations for a reliable assessment of the actual risk to consumers. This requires the availability of a simple and rapid sample preparation protocol providing reliable toxicity data by the CBA-N2a during the first screening assay of the two-tiered approach recommended by ciguatera experts [11]. Although it would have been ideal to have a single optimized protocol further applicable to both functional (e.g., CBA-N2a) and chemical (e.g., LC-MS/MS) screening tests, the present results highlight the difficulty, if not impossibility, to achieve such a goal given the significant differences in methodology and the related matrix effects inherent to these two methods.

## 4. Materials and Methods

### 4.1. Chemicals

Chemicals used for extraction, purification, sample resuspension and CBA-N2a were acetone (AnalaR Normapur, ACS, VWR), citric acid (ACS, VWR), acetonitrile (LiCrosolv, MERCK), ammonium acetate (LC-MS grade, Sigma Aldrich, Saint Louis, MO, USA), chloroform (HPLC-grade Fischer Scientific, Hampton, NH, USA), cyclohexane (AnalaR Normapur, ACS, VWR), dichloromethane (CH_2_Cl_2_) (AnalaR Normapur, ACS, VWR), dimethylsulfoxide (DMSO) (HPLC grade, PanReac Applichem (Darmstadt, Germany), VWR), ethyl acetate (EtOH) (Chromanorm HPLC, Prolabo), hexane (AnalaR, BDH), sodium chloride (NaCl) (AnalaR Normapur, ACS, VWR), methanol (MeOH) (AnalaR Normapur, ACS, VWR) and sodium carbonate (Na_2_CO_3_) (AnalaR Normapur, ACS, VWR).

### 4.2. CTX Standards

Both CTX1B and CTX3C standards were obtained from the Institut Louis Malardé (ILM) CTX standards bank. The CTX3C standard originates from in vitro cultures of the highly toxic *Gambierdiscus polynesiensis* TB92 strain, as described in Chinain et al. [57]. The CTX1B standard originates from a large stock purified from moray eels’ livers, as described in previous studies [65,66,67]. Both standards were resuspended in MeOH to prepare stock solutions at 1 µg mL^−1^.

### 4.3. Biological Materials

The evaluation of the CTX3C extraction/purification efficiencies of the six different protocols was performed using the same carnivorous fish specimen, the Tuamotu emperor (*Lethrinus atkinsoni*, Lethrinidae). This fish was harvested in 2017 from the lagoon of Mangareva Island (Gambier Archipelago, French Polynesia) and had previously shown no CTX-like activity with the CBA-N2a.

For the development of a new, improved extraction/purification protocol, the same batch of two carnivorous fish specimens belonging to the same species, i.e, the bluefin trevally (*Caranx melampygus*, Carangidae), mixed together was used. These two fish were harvested in 2013 and 2014 from the lagoon of Tikehau and the Tubuai Islands (Tuamotu and Australes Archipelagoes, French Polynesia) and had previously shown no CTX-like activity using the CBA-N2a.

To confirm the extraction performance of the newly improved OP protocol, six naturally contaminated fish and shellfish flesh samples (as previously confirmed by the CBA-N2a) were used: giant moray (*Gymnothorax javanicus*, Muraenidae); longface emperor (*Lethrinus olivaceus*, Lethrinidae); bluefin trevally (*Caranx melampygus*); steephead parrotfish (*Chlorurus microrhinos*, Scaridae); yellowfin surgeonfish (*Acanthurus xanthopterus*, Acanthuridae); and trochus gastropod (*Tectus niloticus*, Tegulidae). Finally, for the evaluation of the OP protocol for subsequent LC-MS/MS analyses, a marbled grouper (*Epinephelus polyphekadion*, Serranidae) fish flesh sample was used in addition to the same bluefin trevally (*Caranx melampygus*) and steephead parrotfish (*Chlorurus microrhinos*) samples used for the CBA-N2a analyses.

### 4.4. Spiking Procedure

Aliquots containing 10 g of ground wet non-toxic fish flesh were spiked with 5 µL of a CTX stock solution (corresponding to 5 ng of CTX) and then lyophilized for 48 hours (primary dessication: 44 h, 1 mbar, −20 °C; secondary dessication: 4 h 0.01 mbar, −76 °C) (Martin Christ, Beta 1–8 LDplus, Osterode am Harz, Germany).

### 4.5. Evaluation of Efficiencies of Six Different Protocols for Extraction/Purification of CTX3C

The efficiencies of the six different protocols for the extraction/purification of CTX3C were evaluated using a CBA-N2a.

For each protocol, three aliquots of 10 g wet weight of fish flesh (*Lethrinus atkinsoni*) were spiked with 5 ng of the CTX3C standard. An additional aliquot of 10 g wet weight of fish flesh (non-spiked sample) was also prepared to evaluate matrix effects using the CBA-N2a.

The samples were subjected to six different protocols inspired by some of the protocols commonly used for CTX analyses in fish matrices [23,25,27,45,56]. These six protocols are summarized in Table 3 and detailed in the Supplementary Materials. Additional washing fractions were added to each SPE purification step to ensure that all the CTXs were recovered under the conditions used in the protocols. The fractions obtained along the six chemical procedures were dried and weighted for further analyses by the CBA-N2a to assess CTX3C recovery.

### 4.6. Development of a New, Improved Extraction/Purification Protocol

Based on the advantages of each of the six protocols previously evaluated, numerous tests were performed on aliquots of 10 g wet weight of fish flesh (*Caranx melampygus*) spiked with CTX1B or CTX3C, especially to optimize the solvent mixtures used for the extraction step, leading to the development of the improved OP protocol described below.

#### 4.6.1. Extraction

Ten grams wet weight of spiked (n = 3) and non-spiked (n = 1) fish flesh samples were aliquoted into a 50 mL plastic tube and freeze-dried for 48 h (freeze-dried dry weight approx. 2.3 g). Then, 20 mL of MeOH/H_2_O 60:40 (v:v) was added to the tube. The extraction cycle was homogenization by vortex for 1 min, extraction in a 37 KHz ultrasound bath for 30 min, homogenization by vortex for 1 min before a second extraction cycle in the ultrasound bath for 30 min and finally homogenization by vortex for 1 min.

The tube was then placed in a freezer at −20 °C for 15 min to facilitate fat precipitation. The supernatant was obtained by centrifugation for 10 min at 2800× *g* and transferred to a new 50 mL plastic tube, which was placed in the freezer at −20 °C.

A second and a third extraction cycle were carried out in the same way using 10 mL of MeOH/H_2_O 60:40 and 10 mL of MeOH/H_2_O 80:20, respectively.

The three supernatants were combined in the same tube and 3.5 mL of water was added to bring the MeOH concentration below 60%. The combined supernatants were placed in a freezer at −20°C for 1 h.

#### 4.6.2. Liquid–Liquid Partitioning

A centrifugation step at 2800× *g* for 10 min was then carried out to remove the fats and the resulting supernatant was transferred to a glass separating funnel. A volume of CH_2_Cl_2_ equivalent to twice the volume of supernatant was added to the separatory funnel (approximately 60 mL), and the mixture was mixed gently and then left to settle overnight. The CH_2_Cl_2_ phase was recovered and evaporated under gentle nitrogen flux.

#### 4.6.3. SPE Fractionation

A Sep-Pak aminopropyl (NH2) classic cartridge (360 mg, Waters, Saint-Quentin-En-Yvelines, France) was conditioned with 7 mL CH_2_Cl_2_. The dry extract was resuspended in 2 + 1 + 1 mL of CH_2_Cl_2_ and loaded on the SPE phase. An additional milliliter of CH_2_Cl_2_ was used to rinse the sample tube and was added on the SPE phase. Washing was then performed with 2 mL CH_2_Cl_2_ (F1 fraction). Elution was carried out with 7 mL CH_2_Cl_2_/MeOH 90:10 and the resulting fraction (F2 fraction) was dried under gentle nitrogen flux and weighted for further analyses by the CBA-N2a to assess CTX1B and CTX3C recoveries.

### 4.7. Application of the OP Protocol to Naturally Contaminated Marine Product Samples

To confirm the efficiency of the new, improved OP protocol, tests were performed on six naturally contaminated shellfish and herbivorous and carnivorous fish samples collected from French Polynesian ciguateric areas. For comparison purposes, two to three aliquots of 10 g wet weight of ground marine specimen flesh were extracted using either the OP protocol or protocol #1 (inspired by Darius et al. [68]). The fractions of interest (i.e., the F2 fraction eluted from an NH2 SPE cartridge with CH_2_Cl_2_/MeOH 90:10 for the OP protocol, and the F2 fraction eluted from a C18 SPE cartridge with MeOH/H_2_O 90:10 for protocol #1) were then analyzed with the CBA-N2a.

### 4.8. Neuroblastoma Cell-Based Assay (CBA-N2a)

All CBA-N2a experiments were conducted using N2a, a mouse neuroblastoma cell line (CCL-131) purchased from the American Type Culture Collection (ATCC, Manassas, VA, USA), according to the optimized protocol published by Viallon et al. [13].

#### 4.8.1. CBA-N2a Procedure

Briefly, a density of 50,000 N2a cells per well (total volume 200 µL) was seeded in 5% fetal bovine serum (FBS) RPMI-1640 supplemented medium in 96-well microtiter plates and kept at 37 °C with a 5% CO_2_ humidified atmosphere. The confluence of the cells was reached after 24 h of growth and the culture medium was then removed. The renewal of the culture medium consisted in the deposit of 200 µL of the 2% FBS RPMI-1640 supplemented medium in the upper half of the 96-well plate (OV- condition) and 200 µL of the 2% FBS RPMI-1640 supplemented medium containing a non-destructive treatment of ouabain–veratridine (OV) at [70/7–100/10] µM in the lower half of the 96-well plate (OV+ condition). Then, 10 µL of eight-point serial 1:2 dilutions of the dry extract or CTX standards was added to wells in triplicates for incubation overnight. Cell viability was measured using the methylthiazolyldiphenyl–tetrazolium bromide (MTT) assay after 45 min of incubation at 570 nm using a plate reader (iMark Microplate Absorbance Reader, BioRad, Marnes la Coquette, France). The net absorbance data were fitted to a sigmoidal dose–response curve (variable slope) based on a four-parameter logistic model (4PL). Half-maximal effective concentration (EC_50_) values were obtained using Prism v9.0.2 software (GraphPad, San Diego, CA, USA) and converted in ng CTX equivalents (eq.) g^−1^ of wet tissue (flesh) by comparison with the CTX EC_50_ values.

For all experiments, the absorbance values of the viability controls such as the OV- and OV+ control wells (N2a cells alone) as well as the OV- and OV+ quality control (N2a cells exposed to VGSC standards) wells were used to verify the maintenance of N2a viability and the specific detection of CTXs, as described in Viallon et al. [13].

#### 4.8.2. Strategy of Analyses of Fractions

##### Measure of Matrix Effects

Using the non-spiked samples, all the fractions obtained using the six chemical procedures and the OP protocol were weighed and resuspended in at least 100 µL and up to 520 µL of DMSO depending on the quantity of dry extract to be solubilized.

After a 10-fold dilution in culture medium, an eight-point serial 1:2 dilution in RPMI 2% FBS was prepared, and 10 µL of each dilution was added to 200 µL of the OV- and OV+ treatments (triplicate wells) to determine the MCE that did not induce unspecific effects on N2a cell viability for each fraction. The MCE value of each fraction was determined when the concentration induced more than ≥ 20% mortality and was expressed in µg of dry extract mL^−1^. Then, this value was converted in mg eq. wet weight mL^−1^ according to the dry extract weight/fresh weight (DEW/FW) ratio of each fraction [13].

##### Screening of Fractions

The screening of the fractions from the spiked or naturally contaminated fish samples (n = 2 to 3 according to protocols) was undertaken by the MCE value tested in OV- and OV+ conditions (triplicate wells) to differentiate between the fractions detected with a percentage of viability ≥ 80% in both conditions (classified as negative), those found with a percentage of viability between 20% and 80% in OV- and OV+ (classified as doubtful) and those found with a percentage of viability ≤ 20% in OV+ condition only (classified as positive).

##### Quantification

Quantification of the doubtful fractions was undertaken by testing eight-point serial dilutions under OV- and OV+ conditions (triplicate wells). The concentration range was run from the MCE to check whether the fraction tested induced identical OV- and OV+ mortality due to an excess of dry extract at the MCE limit (false positive).

For positive fractions, the serial dilutions were performed under OV+ conditions only (triplicate wells). The concentration range was run from the MCE and then adjusted according to the toxicity of the fish to obtain a complete sigmoidal dose–response curve.

For each positive fraction, three independent CBA-N2a experiments were run on different days and were expressed in ng CTX3C equivalent g^−1^ of fresh tissue (flesh) for the spiked fish samples (see Section 4.5 and Section 4.6) and herbivores (see Section 4.7) or in ng CTX1B equivalent g^−1^ of fresh tissue (flesh) for the spiked fish samples (see Section 4.6) and carnivores (see Section 4.7).

Coefficients of variation (CVs) were also calculated according to the following formula:

CV (%) = (SD/mean) × 100.

### 4.9. Evaluation of the OP Protocol for Subsequent LC-MS/MS Analyses

Fortification experiments were performed by adding two mixes of CTX1B, CTX3B, CTX3C and CTX4A, each standard at a final concentration of 5 or 10 ng mL^−1^, to the final fractions of interest purified from two carnivorous and one herbivorous fish species according to either the OP protocol, protocol #1 or the Murray et al. [27] protocol (from which protocol #2 was inspired). Pacific CTX reference materials were obtained from the ILM CTX standards bank.

The liquid chromatography–tandem mass spectroscopy (LC-MS/MS) method previously described by Murray et al. [27] for Pacific CTXs was then performed on a Waters Xevo TQ-S triple quadrupole mass spectrometer coupled to a Waters Acquity UPLC i-Class (Waters, Milford, MA, USA). Chromatographic separation was performed using a Waters Acquity UPLC BEH phenyl column (1.7 μm, 100 × 2.1 mm) held at 50 °C. The column was eluted at 0.55 mL min^−1^ with Milli-Q water (A) and 95% acetonitrile (B) mobile phases, each containing 0.2% (*v*/*v*) of a 25% ammonium hydroxide solution (final concentration of 26.7 mM ammonia). Fresh mobile phases were prepared daily to ensure optimal sensitivity and stable retention times. The initial solvent composition was 25% B with a linear gradient to 35% B at 2.0 min, which ramped up to 50% B at 2.5 min, followed by a linear gradient to 75% B at 6.5 min, which ramped up to 95% B at 7.0 min and was maintained at 95% B between 7.0 and 8.0 min. The column was re-equilibrated with 25% B between 8.0 and 9.0 min. The autosampler rack chamber was maintained at 10 °C and the injection volume was 2 μL for the fish extracts.

For the mass spectrometer, the electrospray ionization source was operated in positive-ion mode to monitor the CTXs with the following parameters: capillary voltage 3.5 kV, cone voltage 30 V, source temperature 150 °C, nitrogen gas desolvation flowrate 1000 L h^−1^ at 600 °C, cone gas 150 L h^−1^ and collision cell operated with 0.15 mL min^−1^ argon.

Multi-reaction monitoring (MRM) transitions for the various CTXs were determined from infusion experiments of the toxin standard into the ammoniated mobile phase. Collision energies were optimized for each compound monitored. For quantitative analysis, the following MRM transitions were used, with confirmation transitions shown in parentheses: CTX3B/CTX3C: m/z1023.6 > 155.1 (*m*/*z* 1023.6 > 125.1); CTX4A/CTX4B: *m*/*z* 1061.6 > 155.1 (*m*/*z* 1061.6 > 125.1). For CTX1B quantitation, a total ion chromatogram was generated from the following MRM and pseudo-MRM transitions: *m*/*z* 1128.6 > 95.0, *m*/*z* 1128.6 > 109.0 and *m*/*z* 1133.6 > 1133.6. A dwell time of 20 ms was used. Linear five-point calibrations (0.5–10 ng mL^−1^) of CTX1B, CTX3B, CTX3C and CTX4A were used for quantitation, with coefficients of determination >0.98.

Data acquisition and processing was performed with MassLynx and TargetLynx software V4.1 (Waters, Milford, MA, USA), respectively. Peak areas were integrated and sample concentrations calculated from linear calibration curves generated from external calibration solutions.

## 5. Conclusions

In conclusion, this study provides a comprehensive evaluation of the behavior of nonpolar CTXs, demonstrating large discrepancies in the recoveries in fractions obtained with six different protocols inspired by studies available in the literature. An optimized extraction protocol was then developed in this study enabling recoveries above 70% for both the polar CTX1B and the nonpolar CTX3C in a single fraction of interest, representing a significant methodological improvement in terms of accuracy and precision. This simple protocol is cost-effective, requiring only a single solid-phase extraction cartridge and three days to prepare. Moreover, this optimized protocol demonstrates reliability and efficiency, ensuring consistent results in both naturally contaminated herbivorous and carnivorous marine specimens. The matrix effects observed with CBA-N2a were reduced by a factor >3, allowing for the analysis of larger quantities of the fresh weight matrix. Most importantly, this improved the sensitivity of the detection method, which now meets the very low guidance safety limits recommended by the US-FDA and EFSA. This optimized method is suitable for processing a large number of specimens and advances the field of CTX extraction and detection. By enabling a better assessment of the risks of ciguatera poisoning in marine products, the present study represents a key step in protecting public health and supporting initiatives to monitor marine products for CP risk.

## Figures and Tables

**Table 1:** CTX recoveries reported in the literature and estimated after extraction/purification of fish flesh spiked with CTX standards and subsequent analysis of the final fraction of interest considered as enriched in CTXs.

References	Detection Method	Tested Marine Products	Details of Spiking	Polar CTX Recovery (%)	Nonpolar CTX Recovery (%)
[41]	MBA	3 carnivorous fish species	500 ng CTX1B in 100 g of flesh	CTX1B = 33–85	NT ^c^
[42]	LC-MS/MS	1 carnivorous fish species	0.2 ng CTX1B in 2 g of flesh	CTX1B = 85	NT
[43]	LC-MS/MS	10 carnivorous fish species	3 ng CTX1B in 2 g of flesh	CTX1B = 27–75	NT
[44]	LC-MS/MS	17 carnivorous fish species	20 ng CTX1B in 5 g of flesh	CTX1B = 49–85	NT
[45]	LC-MS/MS	4 carnivorous and 2 herbivorous fish species, 2 invertebrate species	100 pg CTX1B, 500 pg CTX2 ^a^, 500 pg CTX3 ^b^ in 2.5–5 g of flesh	CTX1B = 73–87 CTX2 = 68–83 CTX3 = 61–78	NT
[46]	LC-MS/MS	1 carnivorous fish species	Not specified	CTX1B = 26	NT
[27]	LC-MS/MS	1 carnivorous fish species	0.068, 0.341 and 0.682 µg/kg CTX1B in 5 g of flesh	CTX1B = 44	CTX3C = 13
[47]	ELISA	1 carnivorous fish species	100 pg CTX1B in 10 g of flesh	CTX1B = 32	NT
[23]	LC-MS/MS	2 carnivorous fish species	0.1 µg of CTX1B or CTX3C per kg of fish flesh	CTX1B = 87–96	CTX3C = 105–107
[24]	LC-MS/MS	2 carnivorous and 1 herbivorous fish species	Not specified	CTX1B = 39–62 CTX2 = 35–66 CTX3 = 35–69	CTX3C = 25–35
[48]	LC-MS/MS	Carnivorous fish species	0.1 ng of CTX1B, CTX2 or CTX3 in 5 g of fish flesh	CTX1B = 80–89 CTX2 = 79,80 CTX3 = 71–84	NT
[49]	CBA-N2a	2 carnivorous fish species	Not specified	CTX1B = 32–42	NT

^a^ 52-epi-54-deoxyCTX1B; ^b^ 54-deoxyCTX1B; ^c^ NT: not tested.

**Table 2:** Results of CTX3C spiking experiments (5 ng spiked in 10 g wet weight of flesh) as estimated by CBA-N2a.

Protocol ID#	Fractions Analyzed	Dry Extract (mg)	MCE (mg Equivalent Wet Weight of Fish Flesh mL^−1^)	Recovered CTX3C (ng) ^c^	CTX3C Recovery (%)	Coefficient of Variation (%)
#1	F2 ^a^	2.9	34	1.08 ± 0.16	21.5 ± 3.1	14.5
	F3	10.3	39	0.53 ± 0.10	10.7 ± 1.9	17.8
	Total			1.61 ± 0.18	32.2 ± 3.5	11.0
#2	F1	3.9	NR [>244] ^b^	ND ^d^	-	-
	F2 ^a^	1.2	83	0.32 ± 0.04	6.4 ± 0.9	13.8
	F3	1.4	NR [>343]	ND	-	-
	Total			0.32 ± 0.04	6.4 ± 0.9	13.8
#3	F2	12.9	NR [>184]	ND	-	-
	F1.1	1.4	NR [>343]	ND	-	-
	F1.2 ^a^	1.2	NR [>400]	0.38 ± 0.06	7.6 ± 1.2	16.2
	F1.3	3.7	NR [>270]	0.77 ± 0.16	15.4 ± 3.2	20.7
	Total			1.15 ± 0.21	23.0 ± 4.3	18.7
#4	F4	13.5	NR [>370]	2.11 ± 0.18	42.3 ± 3.5	8.3
	F3.1	0.2	NR [>50]	0.15 ± 0.09	3.0 ± 1.7	55.9
	F3.2 ^a^	0.5	NR [>400]	ND	-	-
	F3.3	0.9	NR [>444]	ND	-	-
	Total			2.27 ± 0.18	45.3 ± 3.7	8.1
#5	F4	26.2	38	0.85 ± 0.20	17.0 ± 4.0	23.3
	F3.2 ^a^	3.3	NR [>145]	ND	-	-
	F3.3	5.2	NR [>92]	ND	-	-
	Total			0.85 ± 0.20	17.0 ± 4.0	23.3
#6	F1.2.1 ^a^	0.4	500	2.04 ± 0.32	40.8 ± 6.3	15.5
	F1.2.2	1.7	NR [>500]	0.13 ± 0.01	2.6 ± 0.2	7.5
	Total			2.17 ± 0.32	43.4 ± 6.4	14.7

^a^ Fractions of interest likely to contain majority of CTXs. ^b^ NR: MCE not reached. In square brackets is indicated the value corresponding to the maximum concentration tested. ^c^ Data represent the mean ± standard deviation (SD) of CTX3C estimated with three CTX3C-spiked fish samples tested in three independent experiments run on different days (n = 9). ^d^ ND: CTX3C not detected.

**Table 3:** Description of the six protocols used for extraction/purification of CTX3C from fish.

Protocol	#1	#2	#3	#4	#5	#6
**Reference**	Darius et al. [68]	Inspired by Murray et al. [27]	Inspired by Sibat et al. [25]	Inspired by Meyer et al. [56] ^a^	Inspired by Mak et al. [45]	Inspired by Nagae et al. [23] ^b^
**Fish flesh amount**	10 g wet	10 g wet	10 g wet and then freeze-dried	10 g wet and then freeze-dried	10 g wet and then freeze-dried	10 g wet
**Extraction**	Acetone 100% ×2 Ultrasonic bath −20 °C precipitation	aq MeOH 60% Blending Ultra-Turrax Heat to 100 °C Ice bath precipitation	Acetone 100% ×2 Ultrasonic bath	MeOH/hexane 73:27 ×2 Blending Ultra-Turrax Washing MeOH	MeOH 100% ×2 aq MeOH 60% Ultrasonic bath Heat to 75 °C	aq MeOH 90% Blending Ultra-Turrax Heat to 80 °C
**Purification 1**	LLP ^c^ aq MeOH 60%/CH_2_Cl_2_ ×2	LLP aq MeOH 60%/CH_2_Cl_2_ ×2	LLP aq MeOH 90%/hexane ×2	LLP aq MeOH 57%/Hexane	SPE C18 Elution aq ACN 65%	LLP aq MeOH 90% + saturated Na_2_CO_3_/hexane
**Purification 2**	LLP aq MeOH 80%/cyclohexane	SPE NH2 Elution CH_2_Cl_2_/MeOH 90:10 (F2 fraction) ^d^	SPE Florisil Elution EtOAc/MeOH 90:10	SPE C18 Elution aq MeOH 80%	LLP aq ACN 65% + 1M NaCl/CH_2_Cl_2_	LLP aq MeOH 90% + saturated Na_2_CO_3_ + 5% citric acid/hexane
**Purification 3**	SPE C18 Elution aq MeOH 90% (F2 fraction) ^d^	/	SPE C18 Elution aq MeOH 90% (F1.2 fraction) ^d^	SPE silica (HILIC mode) Elution acetone/H_2_O 95:5 containing 5 mM ammonium acetate (F3.2 fraction) ^d^	SPE Silica Elution MeOH/chloroform 10:90 (F3.2 fraction) ^d^	SPE Florisil Elution EtOAc/MeOH 85:15
**Purification 4**	/	/	/	/	/	SPE C18 Elution 100% MeOH
**Purification 5**	/	/	/	/	/	SPE PSA Elution aq MeOH 78% (F1.2.1 fraction) ^d^

^a^ Previously adapted from Lewis et al. [42]. ^b^ Previously optimized from Yogi et al. [69]. ^c^ LLP = Liquid–liquid partitioning. **^d^** Fraction of interest to be analyzed for CTX detection/quantification.

## Data Availability

The original contributions presented in this study are included in the article/Supplementary Materials. Further inquiries can be directed to the corresponding authors.

## Supplementary Materials

The following supporting information can be downloaded at https://www.mdpi.com/article/10.3390/md23010042/s1. Supplementary Material: Details of the six protocols used in the present study (inspired by some of the protocols commonly used for CTX analysis from fish matrices) and a schematic representation of the protocols with CTX3C amounts (ng) estimated by CBA-N2a in the testable fractions.
