# Supplementary material for: Optimization of the Extraction Protocol for Pacific Ciguatoxins from Marine Products Prior to Analysis Using the Neuroblastoma Cell-Based Assay
Source: Mar Drugs. 2025 Jan 16;23(1):42. doi: 10.3390/md23010042 (PMC11766842; doi:10.3390/md23010042)
Supplement: Supplementary file 1 [file marinedrugs-23-00042-s001.zip › marinedrugs-3396930-supplementary.pdf]

Article supplementary material

Optimization of the extraction protocol for Pacific ciguatoxins from marine products prior to analysis using the neuroblastoma cell-based assay

Thomas Yon [1], Philippe Cruchet [1], Jérôme Viallon [1], J. Sam Murray [2], Emillie Passfield [2], Mireille Chinain [1], Hélène Taiana Darius [1,*] and Mélanie Roué [3,*]

[1] Institut Louis Malardé (ILM), UMR 241-SECOPOL (IFREMER, ILM, IRD, UPF), Laboratory of Marine Biotoxins, P.O. Box 30, 98713 Papeete, Tahiti, French Polynesia; tyon@ilm.pf (T.Y.); pcruchet@ilm.pf (P.C.); jviallon@ilm.pf (J.V.); mchinain@ilm.pf (M.C.)

[2] Cawthron Institute, 98 Halifax St East, PB 2, The Wood, Nelson 7010, New Zealand; sam.murray@cawthron.org.nz (J.S.M.); emillie.passfield@cawthron.org.nz (E.P.)

[3] Institut de Recherche pour le Développement (IRD), UMR 241-SECOPOL (IFREMER, ILM, IRD, UPF), P.O. Box 6570, 98702 Faa'a, Tahiti, French Polynesia

* Correspondence: tdarius@ilm.pf (H.T.D.); melanie.roue@ird.fr (M.R.)

Supplementary material

Details of the six protocols used in the present study (inspired by some of the protocols commonly used for CTXs analysis from fish matrix) and schematic representation of protocols with CTX3C amounts (ng) estimated by CBA-N2a in testable fractions. Data represent the mean ± standard deviation (SD) (each concentration run in triplicate wells) of three independent experiments run on different days. In bold the CTXs pathway through the different steps (as reported in the literature).

Protocol #1 was performed as previously described in Darius, *et al.* [1]. Briefly, 10 g of fresh flesh was extracted twice in 20 mL acetone under sonication (ultrasonic bath, 37kHz) for 4 h. After centrifugation at 2800 x g for 10 min, both supernatants were stored overnight at 4 °C, centrifuged again at 2800 x g for 10 min, and then dried by rotary evaporation at 40 °C using a rotary evaporator (Rotavapor RII, Büchi, Switzerland). The resulting dried extract was further partitioned between 50 mL CH_2Cl_2 and 25 mL aqueous MeOH 60%, shaken, and allowed to stand for 3 h at room temperature. The resulting aqueous methanolic phase was partitioned a second time with 50 mL CH_2Cl_2, shaken, and left overnight at room temperature. Both resulting CH_2Cl_2 phases were pooled, dried by rotary evaporation, and further defatted by a second overnight partition step with 40 mL cyclohexane and 20 mL aqueous MeOH 80%. The resulting methanolic phase was then evaporated, dried by rotary evaporation, and further purified on a SPE C18 cartridge (360 mg; Waters, USA) preconditioned with 7 mL aqueous MeOH 70%. The sample was resuspended in 4 mL MeOH, then 1 mL of water was added before loading onto the top of the cartridge together with 2 mL aqueous MeOH 70% (resulting in 7 mL aqueous MeOH 70%). The methanolic phase was then washed with 7 mL aqueous MeOH 70% (F1 fraction) and eluted sequentially with 7 mL aqueous MeOH 90% (F2 fraction) and 7 mL pure MeOH (F3 fraction). The resulting fractions were then dried in a SpeedVac concentrator (Savant SPD 1010, Thermo Scientific, USA) and stored at 4°C (Figure S1).

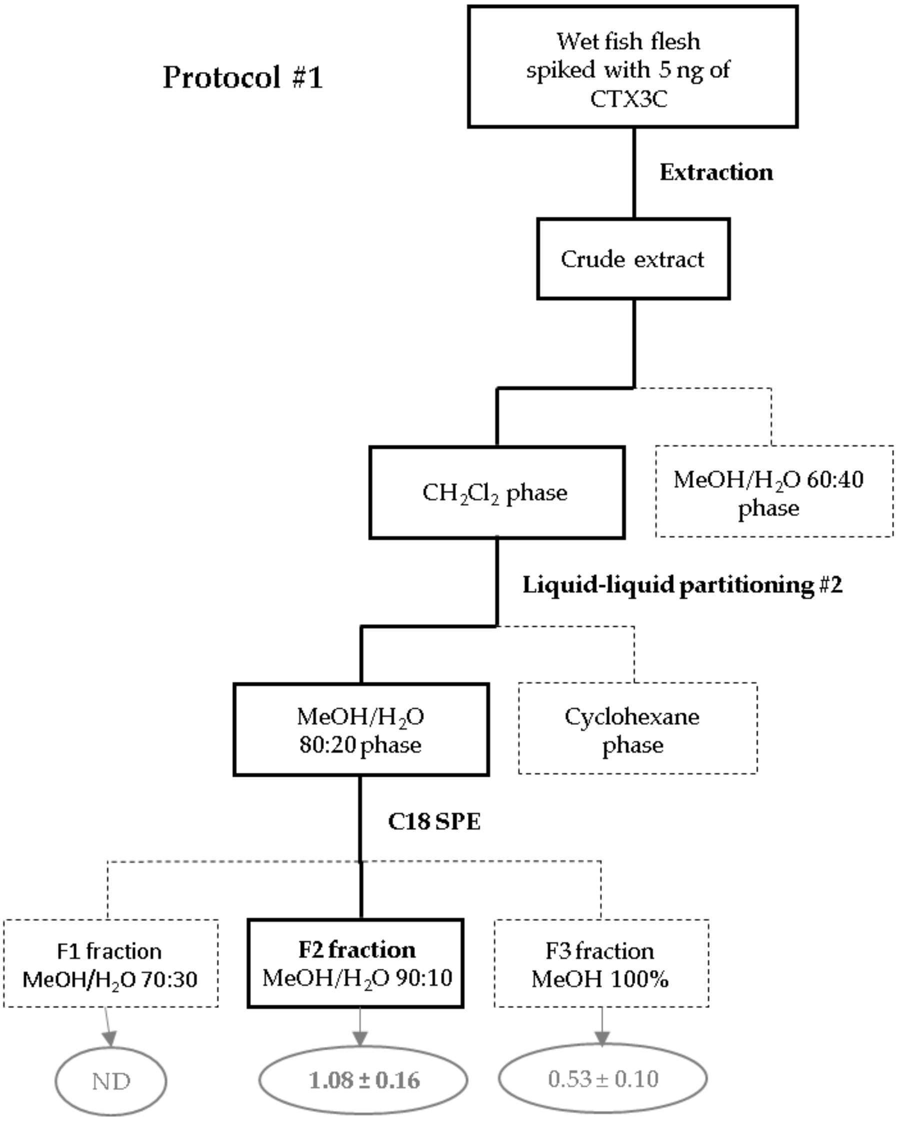

Figure S1. Protocol #1.

Protocol #2 was inspired by Murray, *et al.* [2]. Briefly, 10 g of fresh flesh was homogenized with 20 mL aqueous MeOH 60% using an Ultra-Turrax (IKA, Guangzhou, China) at 17,000 rpm for 1 min. The sample was placed in a 100 °C water bath for 10 min, cooled in an ice bath for 5 min, and then centrifuged at 2800 x g for 10 min. The resulting supernatant (20 mL) was transferred to a 50 mL tube containing 20 mL CH_2Cl_2 for liquid-liquid partition. The sample was thoroughly mixed on a vortex mixer and then centrifuged at 3200 x g for 1 min at 4°C. The upper aqueous MeOH layer and the interphase were aspirated to waste, leaving a 20 mL CH_2Cl_2 phase, which was further dried by rotary evaporation and purified on an aminopropyl (NH_2) SPE cartridge (360 mg; Waters, USA). The column was preconditioned with 7 mL CH_2Cl_2 before loading the extract. The CH_2Cl_2 phase was resuspended in 1 mL CH_2Cl_2 and loaded onto the top of the cartridge, washed with 6 mL CH_2Cl_2 (F1 fraction), and eluted sequentially with 7 mL CH_2Cl_2/MeOH 90:10 (F2 fraction) and 7 mL pure MeOH (F3 fraction). The resulting fractions were then dried under a stream of nitrogen and stored at 4°C (Figure S2).

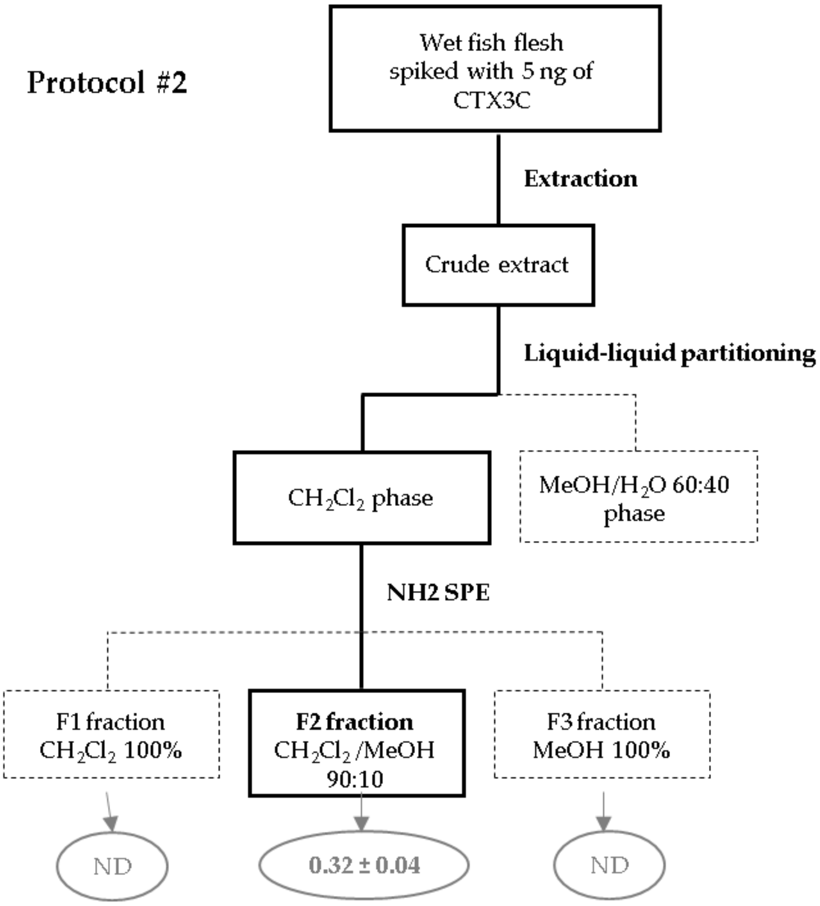

Figure S2. Protocol #2.

Protocol #3 was inspired by Sibat, *et al.* [3]. Briefly, 10 g of fresh flesh was freeze-dried before extraction twice in 30 mL acetone under sonication for 15 min. After centrifugation at 2800 x g for 10 min, the supernatants were dried by rotary evaporation. The residue was reconstituted with 20 mL aqueous MeOH 90% and defatted twice with 40 mL hexane. The methanolic phase was concentrated to dryness by rotary evaporation and then purified using two successive SPE clean-ups. First, a Florisil SPE cartridge (1 g, Waters, USA) was preconditioned with 7 mL ethyl acetate (EtOAc)/MeOH 90:10. The methanolic phase was resuspended in 2 mL EtOAc/MeOH 90:10, loaded onto the top of the cartridge, and eluted sequentially with 6 mL EtOAc/MeOH 90:10 (F1 fraction) and 7 mL pure MeOH (F2 fraction). The resulting fractions were then dried under a stream of nitrogen and stored at 4°C. The F1 fraction was then resuspended in 2 mL aqueous MeOH 60% and loaded onto the top of a C18 SPE cartridge (360 mg, Waters, USA) preconditioned with 3 mL aqueous MeOH 60%. The sample was then washed with 3 mL aqueous MeOH 75% (F1.1 fraction) and eluted sequentially with 6 mL aqueous MeOH 90% (F1.2 fraction) and 6 mL pure MeOH (F1.3 fraction). The resulting fractions were then dried in a SpeedVac concentrator and stored at 4°C (Figure S3).

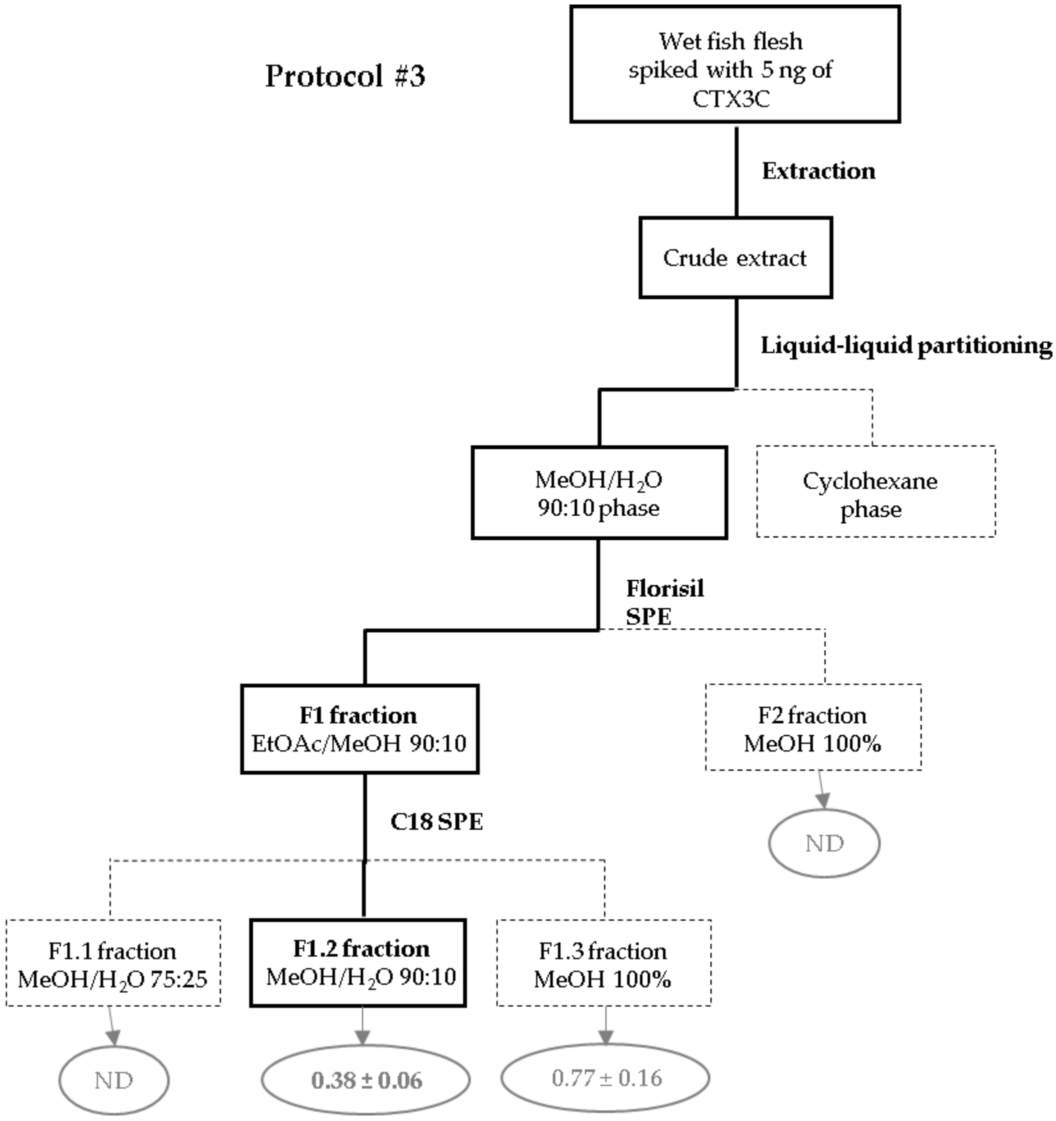

Figure S3. Protocol #3.

Protocol #4 was inspired by Meyer, *et al.* [4], previously adapted from Lewis, *et al.* [5]. Briefly, 10 g of fresh flesh was freeze-dried before being homogenized twice in 8 mL MeOH and 3 mL n-hexane using an Ultra-Turrax at 17,000 rpm for 2 min. After centrifugation at 2800 x g for 10 min, both supernatants were transferred into a separatory funnel. The fish matrix was then washed with 4 mL MeOH by vortexing for 2 min. After centrifugation at 2800 x g for 10 min, the supernatant was transferred to the same separatory funnel and 15 mL H_2O was added for subsequent liquid-liquid partition. The resulting methanolic phase was collected and centrifuged at 2800 x g for 10 min. A C18 SPE cartridge (360 mg, Waters, USA) was sequentially preconditioned with 6 mL MeOH and 6 mL aqueous MeOH 50%. The methanolic phase (35 mL) was loaded directly onto the top of the column (F1 fraction), washed with 6 mL aqueous MeOH 65% (F2 fraction), and eluted sequentially with 12 mL aqueous MeOH 80% (F3 fraction) and 7 mL MeOH (F4 fraction). The resulting fractions were then dried in a SpeedVac concentrator and stored at 4°C. A silica SPE cartridge (1 g, Waters, USA) used in HILIC mode was sequentially preconditioned with 2 mL MeOH and 4 mL acetone/H_2O 95:5 containing 5 mM ammonium acetate. The F4 fraction was then resuspended in 1 mL acetone containing 0.1 % formic acid. An additional wash of the tube containing the F4 fraction was performed with 2 mL acetone, which was added to the formic acid/acetone solution and diluted with 0.3 mL H_2O (resulting in acetone/ H_2O 90:10) before being load onto the top of the column (F3.1 fraction). The sample was then eluted with 8 mL acetone/H_2O 95:5 containing 5 mM ammonium acetate (F3.2 fraction) and 7 mL MeOH (F3.3 fraction). The resulting fractions were then dried under a stream of nitrogen and stored at 4°C (Figure S4).

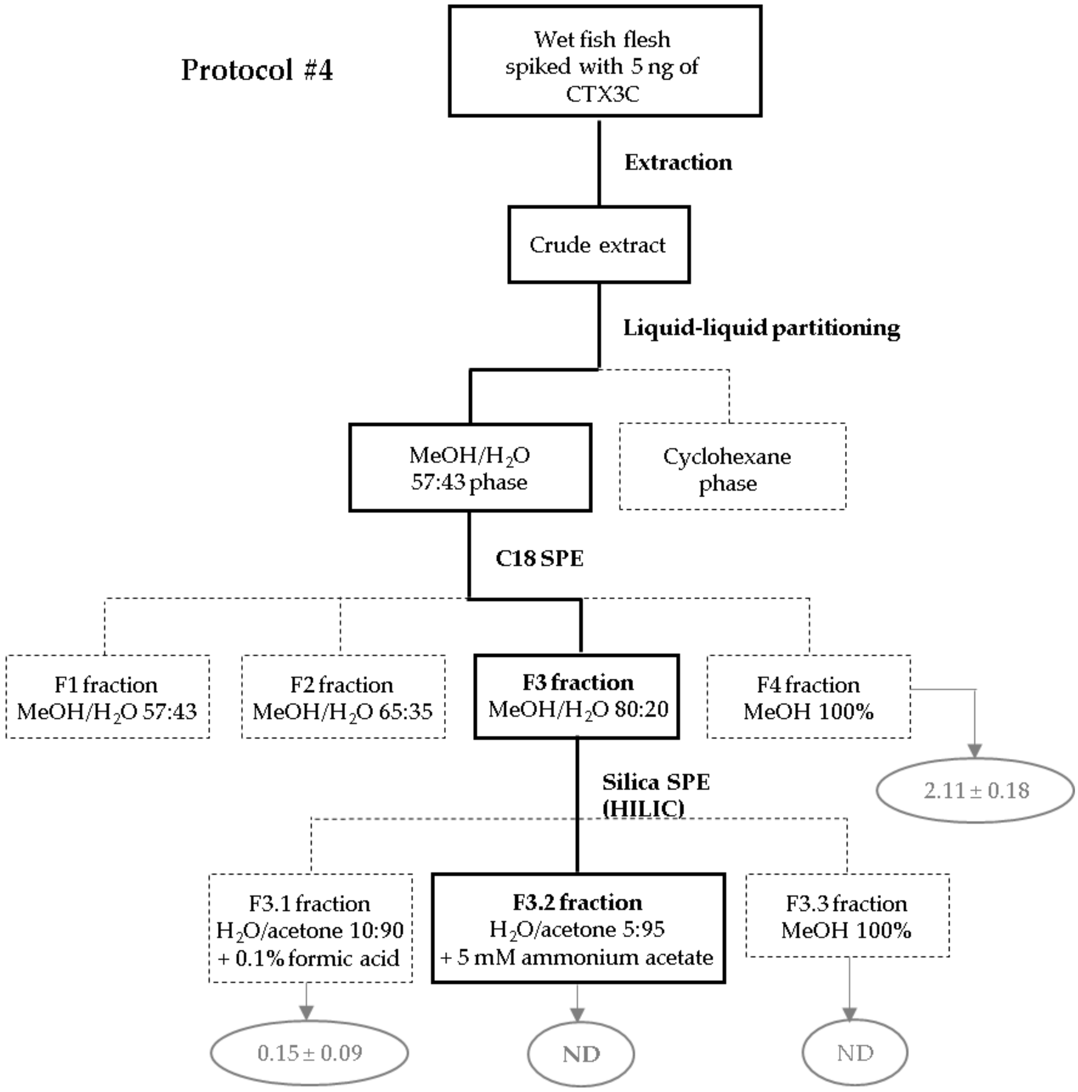

Figure S4. Protocol #4.

Protocol #5 was inspired by Mak, *et al.* [6]. The original protocol used an accelerated solvent extraction (ASE) system for the extraction step, but this material was not available in French Polynesia. Therefore, 10 g of fresh flesh was freeze-dried before sequential extraction in 30 mL MeOH, 20 mL MeOH and 30 mL aqueous MeOH 60% under sonication for 2h and boiling for 10 min in a water bath at 75°C. After centrifugation at 2800 x g for 10 min, the resulting supernatants were pooled and dried by rotary evaporation. Two C18 SPE cartridges (360 mg, Waters, USA) were stacked and sequentially preconditioned with 15 mL acetonitrile, 15 mL pure MeOH, and 10 mL H_2O. The crude extract was resuspended in 11 mL aqueous MeOH 50% and loaded onto the top of the column (F1 fraction). The sample was then washed with 6,5 mL aqueous MeOH 65% (F2 fraction) and eluted sequentially with 12 mL acetonitrile (F3 fraction) and 8 mL pure MeOH (F4 fraction). The resulting F3 fraction was then partitioned with 6 mL chloroform and 6 mL of a 1 M NaCl solution. The chloroform phase was dried under a stream of nitrogen and purified on a silica SPE cartridge (1 g; Waters, USA) preconditioned with 30 mL chloroform. The sample was reconstituted in 2,5 mL chloroform/MeOH 98:2, loaded onto the top of the cartridge (F3.1 fraction), and eluted sequentially with 8 mL chloroform/MeOH 90:10 (F3.2 fraction) and 8 mL pure MeOH (F3.3 fraction). The resulting fractions were then dried under a stream of nitrogen and stored at 4°C (Figure S5).

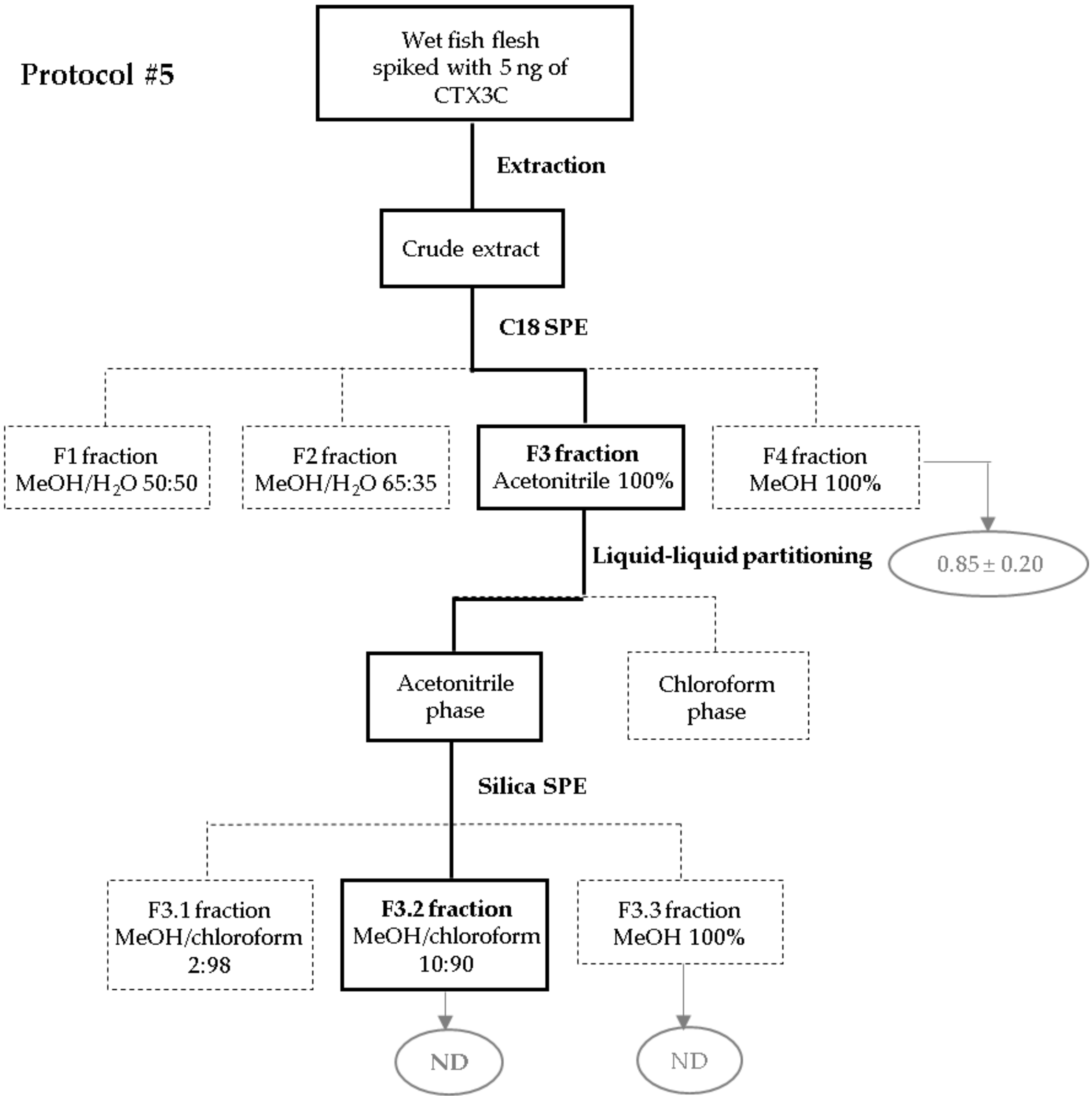

Figure S5. Protocol #5.

Protocol #6 was inspired by Nagae, *et al.* [7], previously optimized from Yogi, *et al.* [8]. Briefly, 10 g of fresh flesh was homogenized with 190 mL aqueous MeOH 90% using an Ultra-Turrax at 17,000 rpm. The sample was placed in an 80 °C water bath for 10 min and then centrifuged at 2800 x g for 10 min. 150 mL of the resulting supernatant was combined with 2 mL of a saturated Na_2CO_3 solution and 100 mL hexane for partitioning. After centrifugation at 2800 x g for 10 min, the methanolic phase was combined with 10 mL of a 5% citric acid solution and 200 mL of hexane in a separatory funnel for partitioning. The resulting methanolic phase was dried by rotary evaporation and purified by three successive SPE clean-ups. First, a Florisil SPE cartridge (plus long, 910 mg, Waters, USA) was preconditioned with 10 mL hexane/EtOAc/H_2O 1:1:0.5. The methanolic phase was resuspended in 7.5 mL hexane/EtOAc/H_2O 1:1:0.5, loaded onto the top of the cartridge, and eluted sequentially with 10 mL EtOAc/MeOH 85:15 (F1 fraction) and 10 mL pure MeOH (F2 fraction). The resulting fractions were dried under a stream of nitrogen and stored at 4°C. The F2 fraction was then resuspended in 6 mL aqueous MeOH 70% and loaded onto the top of two stacked C18 SPE cartridges (360 mg, Waters, USA) preconditioned with 8 mL aqueous MeOH 70%. The sample was then washed with 2 mL aqueous MeOH 70% (F1.1 fraction) and eluted with 8 mL pure MeOH (F1.2 fraction). The resulting fractions were dried dried in a SpeedVac concentrator (Savant SPD 1010, Thermo Scientific, USA) and stored at 4°C. Finally, the F1.2 fraction was then resuspended in aqueous MeOH 78% and loaded onto the top of a primary and secondary amine (PSA) SPE cartridge (360 mg, Waters, USA) preconditioned with 8 mL aqueous MeOH 78%. The sample was then sequentially eluted with 8 mL aqueous MeOH 78% (F1.2.1 fraction) and 8 mL pure MeOH (F1.2.2 fraction). The resulting fractions were dried in a SpeedVac concentrator and stored at 4°C (Figure S6).

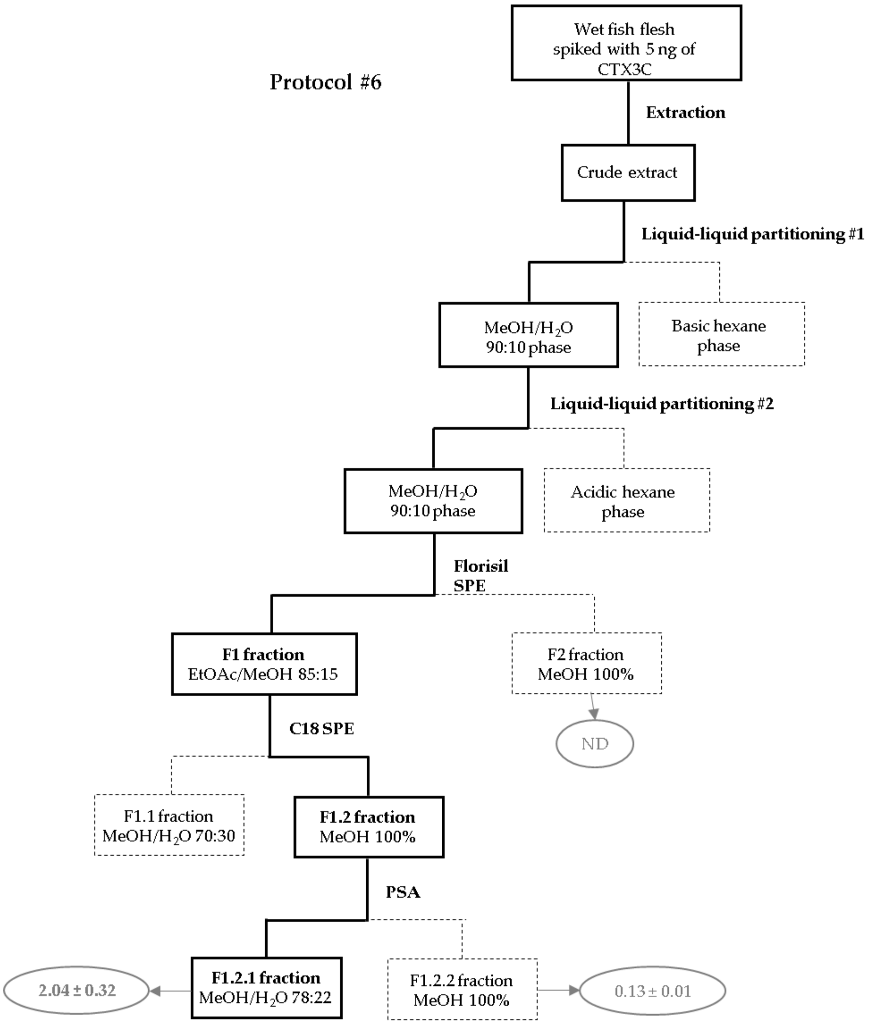

Figure S6. Protocol #6.

References

1. Darius, H. T.; Revel, T.; Viallon, J.; Sibat, M.; Cruchet, P.; Longo, S.; Hardison, D. R.; Holland, W. C.; Tester, P. A.; Litaker, R. W.; McCall, J. R.; Hess, P.; Chinain, M., Comparative Study on the Performance of Three Detection Methods for the Quantification of Pacific Ciguatoxins in French Polynesian Strains of Gambierdiscus polynesiensis. *Marine drugs* **2022,** 20, (6), 348.
2. Murray, J. S.; Boundy, M. J.; Selwood, A. I.; Harwood, D. T., Development of an LC-MS/MS method to simultaneously monitor maitotoxins and selected ciguatoxins in algal cultures and P-CTX-1B in fish. *Harmful Algae* **2018,** 80, 80-87.
3. Sibat, M.; Herrenknecht, C.; Darius, H. T.; Roué, M.; Chinain, M.; Hess, P., Detection of pacific ciguatoxins using liquid chromatography coupled to either low or high resolution mass spectrometry (LC-MS/MS). *Journal of Chromatography A* **2018,** 1571, 16-28.
4. Meyer, L.; Carter, S.; Capper, A., An updated ciguatoxin extraction method and silica cleanup for use with HPLC-MS/MS for the analysis of P-CTX-1, PCTX-2 and P-CTX-3. *Toxicon* **2015,** 108, 249-256.
5. Lewis, R. J.; Yang, A. J.; Jones, A., Rapid extraction combined with LC-tandem mass spectrometry (CREM-LC/MS/MS) for the determination of ciguatoxins in ciguateric fish flesh. *Toxicon* **2009,** 54, (1), 62-66.
6. Mak, Y. L.; Wu, J. J.; Chan, W. H.; Murphy, M. B.; Lam, J. C. W.; Chan, L. L.; Lam, P. K. S., Simultaneous quantification of Pacific ciguatoxins in fish blood using liquid chromatography-tandem mass spectrometry. *Analytical and Bioanalytical Chemistry* **2013,** 405, (10), 3331-3340.
7. Nagae, M.; Igarashi, T.; Mizukoshi, K.; Kuniyoshi, K.; Oshiro, N.; Yasumoto, T., Development and Validation of an LC-MS/MS Method for the Ultra-Trace Analysis of Pacific Ciguatoxins in Fish. *Journal of AOAC INTERNATIONAL* **2021,** 104, (5), 1272-1281.
8. Yogi, K.; Sakugawa, S.; Oshiro, N.; Ikehara, T.; Sugiyama, K.; Yasumoto, T., Determination of Toxins Involved in Ciguatera Fish Poisoning in the Pacific by LC/MS. *J AOAC Int* **2014,** 97, (2), 398-403.
